# Higher Dietary Antioxidant Index Is Associated with Better Lipid Profile in Women with Coronary Artery Disease

**DOI:** 10.3390/healthcare14081085

**Published:** 2026-04-19

**Authors:** Mariana Moya-García, Wendy Campos-Pérez, Mariana Pérez-Robles, Sissi Godínez-Mora, Sarai Citlalic Rodríguez-Reyes, Liliana Estefanía Ramos-Villalobos, Erika Martínez-López

**Affiliations:** 1 Instituto de Nutrigenética y Nutrigenómica Traslacional, Centro Universitario de Ciencias de la Salud, Universidad de Guadalajara, Guadalajara 44100, Jalisco, Mexico; mariana.moya8125@alumnos.udg.mx (M.M.-G.); wendy.campos4381@academicos.udg.mx (W.C.-P.); mariana.perez@academicos.udg.mx (M.P.-R.); sissi.godinez5582@alumnos.udg.mx (S.G.-M.); citlalic.rodriguez@academicos.udg.mx (S.C.R.-R.); 2 Servicio de Cardiología del Hospital Civil de Guadalajara “Dr. Juan I. Menchaca”, Guadalajara 44340, Jalisco, Mexico; liliana.ramos5375@academicos.udg.mx

**Keywords:** coronary artery disease, dietary antioxidant index, phase angle, antioxidants, HDL, oxidized lipoproteins

## Abstract

Background/Objectives: Cardiovascular diseases are the leading cause of mortality worldwide, with coronary artery disease (CAD) being the most prevalent. An atherogenic diet contributes to oxidative stress by promoting lipid peroxidation in lipoproteins and cellular membranes, thereby compromising membrane integrity, which is reflected in lower phase angle (PhA) values. Dietary antioxidants play a crucial role in cellular health and in reducing atherosclerotic risk; therefore, the Dietary Antioxidant Index (DAI) is an important measure, as dietary antioxidants may counteract oxidative damage. This study aimed to assess the association between anthropometric, PhA, and biochemical variables across groups classified according to DAI. Methods: This was an analytical cross-sectional study. A total of 107 subjects, with and without CAD, were included. Oxidized LDL (oxLDL) and oxidized HDL (oxHDL) were determined using the ELISA technique. PhA was measured by bioelectrical impedance analysis, and DAI was calculated using the formula proposed by Wright et al. Results: DAI was positively associated with HDL concentrations in women with CAD, indicating that HDL levels increased by 5.8 mg/dL for each unit increase in DAI (R^2^ = 0.625, *p* = 0.001). Furthermore, for each unit increase in DAI, the TC/HDL ratio decreased by 0.3 (R^2^ = 0.625, *p* = 0.006), and the LDL/HDL ratio decreased by 0.2 (R^2^ = 0.506, *p* = 0.012). Conclusions: A higher DAI is associated with a more favorable lipid profile in women with CAD, particularly with higher HDL concentrations and lower TC/HDL and LDL/HDL ratios.

## 1. Introduction

Cardiovascular diseases (CVD) are the leading causes of death worldwide and in Mexico, with coronary artery disease (CAD) being the most prevalent [1]. Several modifiable factors, such as physical inactivity, overweight or obesity, smoking, alcohol consumption, and unhealthy dietary habits, significantly contribute to the development of CAD [2,3,4]. In particular, a diet characterized by low intake of fruits, vegetables, and whole grains, along with high intake of sodium, added sugars, saturated fats, trans fatty acids, and an imbalance in the *n*–6:*n*–3 ratio of polyunsaturated fatty acids (PUFAs), promotes increased oxidative stress and a higher risk of CAD [2,3]. Oxidative stress is defined as an imbalance between the production of reactive oxygen species (ROS) and endogenous antioxidant capacity [5].

Oxidative stress induces oxidative modifications in the phospholipids of lipoproteins and cellular membranes [5,6]. At the cellular membrane level, lipid peroxidation disrupts the structure and function of the lipid bilayer, compromising its stability and increasing permeability [7]. A key indicator of cellular integrity is the phase angle (PhA), a parameter derived from bioelectrical impedance that reflects membrane functionality and body cell mass [8,9]. Higher PhA values are associated with structurally and functionally intact cells, whereas lower values indicate alterations in membrane structure and function, reflecting a reduced ability to maintain appropriate membrane potential [8,10,11]. Given the critical role of oxidative damage in membrane integrity, dietary factors that modulate oxidative stress are essential for preserving cellular function.

A diet rich in fruits, vegetables, whole grains, lean proteins, fiber, and antioxidants, along with an adequate *n*–6:*n*–3 PUFA ratio, has been shown to exert a protective effect against oxidative stress and inflammation by neutralizing free radicals and acting synergistically with endogenous antioxidant systems [2,5,12,13]. In this context, the Dietary Antioxidant Index (DAI) is a validated tool that assesses the overall antioxidant content of the diet by considering nutrients such as vitamins A, C, and E, as well as zinc, selenium, and manganese [12]. These antioxidants play a key role in reducing oxidative stress, lipid peroxidation and inflammation, thereby helping to slow the progression of atherosclerosis and the development of CAD [2,5,12,13]. Furthermore, higher DAI values have been associated with a lower prevalence and risk of dyslipidemia [6].

To our knowledge, the relationship between antioxidant quality indices (e.g., DAI and PhA) and oxidized lipoproteins has not yet been established. Considering that a higher DAI may reflect a nutritional profile capable of enhancing PhA and attenuating oxidative damage, further research is warranted. This is particularly relevant for the Mexican population and for individuals with CAD. Therefore, this study aimed to evaluate the associations between anthropometric, biochemical, and PhA variables in Mexican subjects stratified according to their DAI values.

## 2. Materials and Methods

### 2.1. Study Subjects

In this analytical cross-sectional study, a total of 107 subjects were included: 51 participants diagnosed with CAD and 56 without a diagnosis of CAD. All participants were recruited in western Mexico between January 2022 and December 2024. Individuals with CAD were enrolled through the Cardiology Service of the Hospital Civil de Guadalajara “Dr. Juan I. Menchaca”, where patients were consecutively recruited during the study period. All individuals meeting the inclusion criteria were invited to participate, including both newly diagnosed and chronic cases of CAD. Participants without CAD were recruited through social media advertisements and were scheduled for a preliminary medical screening to assess eligibility for inclusion in the study.

Inclusion criteria for both CAD and non-CAD groups were an age range of 30–75 years and a body mass index (BMI) ≥ 18.5 kg/m^2^. Participants in the CAD group were required to have a confirmed diagnosis of CAD, whereas individuals in the non-CAD group had no prior diagnosis of the disease. Exclusion criteria for both groups included autoimmune disorders, chronic kidney disease, and cancer. The study was conducted at the Instituto de Nutrigenética y Nutrigenómica Traslacional of the Universidad de Guadalajara.

### 2.2. Medical Screening for Non-CAD Participants

Participants in the non-CAD group were screened using a structured questionnaire based on the Rose Angina Questionnaire, which included items on chest pain characteristics, prior diagnosis of CAD, and medication use. Individuals reporting a history of myocardial infarction, angina, coronary revascularization, or symptoms consistent with angina were excluded to minimize misclassification bias.

### 2.3. Coronary Artery Disease Diagnosis

CAD diagnosis was established by a cardiologist based on the evaluation of ischemic symptoms. In men, typical symptoms included oppressive chest pain radiating to the left arm, retrosternal pain, and dyspnea. In women, more frequent atypical presentations were considered, including fatigue, imbalance, irregular heartbeat, and dyspnea. Biochemical markers were also considered, including elevated creatine kinase levels and sex-specific thresholds for cardiac troponins. Electrocardiographic evidence of ST-segment abnormalities and confirmation of arterial obstruction by coronary angiography were obtained in all participants. Following international guidelines, patients were categorized as having stable angina, unstable angina, ST-segment elevation myocardial infarction, or non-ST-segment elevation myocardial infarction. When participants did not meet criteria for these categories due to nonspecific clinical presentation, they were classified as having mixed CAD. This classification was conducted in accordance with the guidelines of the American College of Cardiology, American Heart Association and European Society of Cardiology.

### 2.4. Pharmacological Therapy

Pharmacological therapy in patients with CAD consisted of lipid-lowering agents (atorvastatin, bezafibrate, pravastatin, ciprofibrate), vasodilators (isosorbide, nifedipine), anticoagulants and antiplatelet agents (acetylsalicylic acid, clopidogrel), antihypertensives (metoprolol, losartan, enalapril) and glucose-control lowering agents (metformin).

### 2.5. Dietary Intake Assessment

Dietary intake data were obtained through interviews conducted by a trained nutritionist using a habitual day food record. Nutrient intake was analyzed using Nutritionist Pro Diet Analysis software v7.9 (Axxya Systems, Stafford, TX, USA), which incorporates data from the U.S. Department of Agriculture, the Canadian Nutrient File, and European Union databases [14].

### 2.6. Calculation of Dietary Antioxidant Index

Dietary intake of vitamin A, vitamin C, vitamin E, selenium, manganese, and zinc was obtained from the habitual day food record. Based on these data, the mean and standard deviation (SD) for each nutrient were calculated. The DAI was computed using the formula proposed by Wright et al. [15], in which each nutrient intake was standardized by subtracting the sample mean from the individual intake and dividing by the corresponding SD [5,12,13]. The final DAI score was obtained by summing the standardized values of all included nutrients (see formula below).

$$\mathrm{DAI}=\sum_{i=1}^{n}\frac{\mathrm{Individual}\;\mathrm{Intake}-\mathrm{Mean}}{\mathrm{SD}}$$

### 2.7. Biochemical Analysis

Venous blood samples were collected from all participants following an 8–10 h fasting period and centrifuged to obtain serum. Serum levels of glucose, total cholesterol (TC), high-density lipoprotein cholesterol (HDL), and triglycerides (TG) were measured using a Vitros 350 dry chemistry analyzer (Ortho-Clinical Diagnostics, New York, NY, USA). Low-density lipoprotein (LDL) and very-low-density lipoprotein (VLDL) concentrations were estimated using the Friedewald formula when TG levels were <400 mg/dL [16]. Lipid ratios (TG/HDL, TC/HDL, and LDL/HDL) were calculated by dividing TG, TC, and LDL values by HDL, respectively.

### 2.8. Quantification of Oxidized Lipoproteins

Serum concentrations of oxidized low-density lipoprotein (oxLDL) and oxidized high-density lipoprotein (oxHDL) were measured using enzyme-linked immunosorbent assay (ELISA) kits (Cell Biolabs; catalog numbers: STA-888 and STA-369, San Diego, SD, USA). Absorbance was measured at 450 nm using a Multiskan SkyHigh microplate reader (Thermo Fisher Scientific, Waltham, MA, USA), and results were expressed in ng/mL.

To calculate oxidized lipoprotein ratios (oxLDL/LDL and oxHDL/HDL), oxidized lipoprotein values were first converted to mg/dL. Ratios were then calculated, and the resulting values were log-transformed using the natural logarithm (ln).

### 2.9. Anthropometric Measurements

Body weight was measured using a seca scale (seca 803, seca GmbH & Co., Hamburg, Germany), and height was measured using a seca stadiometer. Waist circumference (WC) was assessed using a Lufkin Executive Thinline measuring tape 2 mm; (Apex Tool Group, New Brighton, MN, USA). Body fat percentage (BFP) was determined using tetrapolar bioelectrical impedance analysis (BIA) with the Quantum V Segmental device (RJL Systems, Clinton Township, MI, USA). BMI was calculated as weight (kg) divided by height squared (m^2^).

### 2.10. Phase Angle

PhA was assessed using tetrapolar bioelectrical impedance analysis with the Quantum V Segmental BIA device (RJL Systems, Clinton Township, MI, USA). Participants were instructed to fast for 8–10 h, maintain adequate hydration, and remove all metal accessories prior to measurement. Measurements were performed with participants in a supine position, with legs slightly apart and arms positioned away from the torso. Eight adhesive electrodes were placed, two on each hand and foot, near the bony prominences of the wrist and ankle.

### 2.11. Ethical Guidelines

All participants provided written informed consent, and confidentiality was strictly maintained. All procedures were conducted in accordance with the principles of the Declaration of Helsinki. The study was approved by the Research Ethics Committee (Comité de Investigación, Ética en Investigación y de Bioseguridad) of the Centro Universitario de Ciencias de la Salud, Guadalajara, Jalisco, Mexico (registration number 2020–2024: CI-08320, approved on 27 October 2020; amendment 2024–2028: CI-01524).

### 2.12. Statistical Analysis

Sample size was calculated using OpenEpi version 3.0, applying the difference-of-means method, based on a previous study conducted in a Mexican population (2021) that evaluated PhA in relation to diabetes-related complications, resulting in a required sample size of 106 subjects [17].

Statistical analyses were performed using SPSS software v.20 (IBM Corp., New York, NY, USA). A *p-*value < 0.05 and a 95% confidence interval were considered statistically significant. Normality of quantitative variables was assessed using the Shapiro–Wilk test. Age was expressed as mean ± standard deviation. Between-groups comparisons were conducted using a univariate general linear model adjusted for age, sex, alcohol consumption, smoking status, and physical activity. Study groups were categorized based on the median DAI, as no established cut-off points are currently available.

Additionally, linear regression analyses were performed to evaluate the associations between independent variables and the outcome of interest.

## 3. Results

### 3.1. Characteristics of the Study Subjects

**Figure 1:**
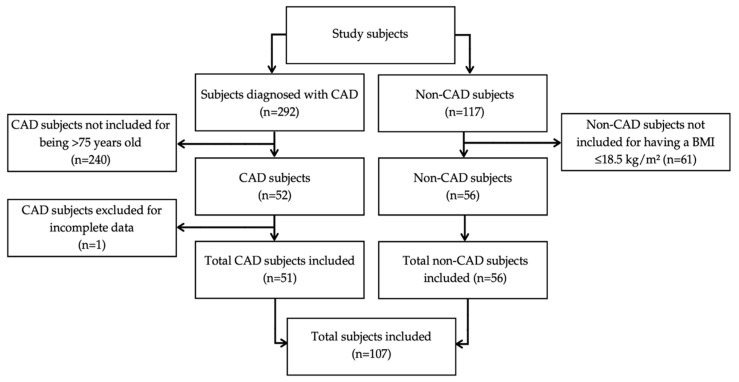
Flow diagram of study subjects.

A total of 107 participants were included in this study (Figure 1), with a mean age of 55.1 ± 12.7 years. Of these, 47.7% (*n* = 51) belonged to the CAD group, which comprised 66.7% men (*n* = 34) and 33.3% women (*n* = 17), with a mean age of 60.2 ± 12.5 years. The remaining 52.3% (*n* = 56) corresponded to the non-CAD group, including 60.7% men (*n* = 34) and 39.3% women (*n* = 22), with a mean age of 50.5 ± 11.1 years. Participants with CAD were significantly older than those in the non-CAD group (*p* < 0.001). No statistically significant differences were observed in sex distribution between groups (*p* = 0.552).

### 3.2. Comparative Analysis of Biochemical and Anthropometric Variables Between Study Groups

The biochemical and anthropometric characteristics of each group, along with between-group comparisons, are presented in Table 1.

Regarding biochemical variables, subjects with CAD showed lower TC and LDL concentrations than those in the non-CAD group (*p* < 0.001). Among men, HDL concentrations were significantly lower in the CAD group (*p* = 0.002). In contrast, the LDL/HDL ratio was significantly lower in women with CAD than in women in the non-CAD group (*p* = 0.035). OxLDL concentrations were higher in the CAD group (*p* = 0.011), whereas both the oxLDL/LDL and oxHDL/HDL ratios were significantly higher in the non-CAD group (*p* < 0.001 and *p* = 0.005, respectively). Higher glucose concentrations were also observed in subjects with CAD compared with non-CAD participants (*p* = 0.021).

Regarding anthropometric variables, WC was significantly higher in women with CAD than in non-CAD women (*p* = 0.016). Likewise, non-CAD men exhibited higher PhA values than men in the CAD group (*p* = 0.005).

### 3.3. Comparative Analysis of Biochemical and Anthropometric Variables According to DAI

Participants were classified according to the median DAI value within each study group. The median DAI was −1.2 for the CAD group and −0.4 for the non-CAD group.

Regarding biochemical variables (Table 2), women with CAD and a DAI < −1.2 presented higher TC/HDL (*p* = 0.001) and LDL/HDL ratios (*p* = 0.001) than those with DAI values above the median.

For anthropometric variables, no significant differences were observed within either study group.

### 3.4. Association Between DAI and Study Variables

Among women with CAD, DAI was positively associated with HDL concentrations (*p* = 0.001), indicating that HDL concentrations increased by 5.8 mg/dL for each unit increase in DAI. In contrast, inverse associations were observed between DAI and the TC/HDL ratio, such that for each unit increase in DAI, the ratio decreased by 0.3 (*p* = 0.006), and between DAI and the LDL/HDL ratio, such that for each unit increase in DAI, the ratio decreased by 0.2 (*p* = 0.012) (Table 3).

In the non-CAD group, a significant association was also observed between DAI and PhA (Table 4). Linear regression analysis showed a positive relationship, indicating that PhA increased by 0.07° for each unit increase in DAI (*p* = 0.006). The same analysis was performed in the CAD group; however, the association was not statistically significant.

## 4. Discussion

Coronary artery disease (CAD) is a multifactorial disease, and it has been reported that in Mexico, 60% of the adult population presents at least one risk factor for its development, indicating its high prevalence. These risk factors should be extensively studied, including novel and non-invasive biomarkers such as oxidized lipoproteins levels and phase angle (PhA) determination [18].

In the present study, the analysis of biochemical variables showed that subjects with CAD had significantly lower concentrations of LDL, TC, and HDL among men. This is consistent with the findings of Fang et al., who reported that subjects with CVD, compared with those without CVD, had lower concentrations of LDL (2.5 ± 0.8 vs. 2.9 ± 0.9 mmol/L, *p* < 0.001), TC (4.6 ± 1.0 vs. 5.0 ± 1.0 mmol/L, *p* < 0.001), and HDL (1.2 ± 0.4 vs. 1.3 ± 0.4 mmol/L, *p* < 0.001) [19]. These findings are also consistent with those reported by Ma et al., in which subjects with CAD, compared with those without CAD, had lower concentrations of LDL (2.5 vs. 3.0 mmol/L, *p* < 0.001), TC (4.6 vs. 5.0 mmol/L, *p* < 0.001), and HDL (1.2 vs. 1.3 mmol/L, *p* < 0.001) [5].

These lipid profile findings may be explained by the pharmacological treatments received by patients with CAD, particularly lipid-lowering medications, mainly atorvastatin, which is effective in reducing LDL concentrations but has a limited effect on HDL levels [1,18]. An additional important consideration in this study is the significant age difference between the CAD and non-CAD groups, with CAD participants being older. Although the analyses were adjusted for age, it is well established that age is strongly associated with lipid metabolism and cardiovascular risk [20].

The finding of significantly higher LDL and TC concentrations, as well as LDL/HDL ratio, among non-CAD subjects represents an important observation, as these are recognized risk factors for the development of atherosclerosis and CAD. In this regard, dyslipidemia has been reported as the main cardiovascular risk factor in Mexico, increasing the risk of developing CAD by up to 64.6%, given that LDL can undergo oxidation and become retained in the arterial wall, thereby promoting its accumulation in the intima [2,21,22,23].

Although the measurement of oxidized lipoproteins is not commonly included in standard clinical evaluations, their determination represents important added value, as these biomarkers provide more specific insight into the oxidative processes involved in atherosclerotic progression. Therefore, recent CAD research has highlighted the need to evaluate these biomarkers in populations at increased risk, as was done in the present study [24,25].

In this context, significantly higher concentrations of oxLDL were observed in the CAD group. This finding is clinically relevant because, as previously reported by our research group, oxLDL values ≥ 7358.8 ng/mL are associated with an increased risk of recurrent ischemic events and multivessel CAD [1], suggesting that these subjects may remain at elevated cardiovascular risk despite previous diagnosis or treatment.

Oxidized lipoprotein ratios were also included because they reflect the relative degree of lipoprotein oxidation rather than their absolute concentrations. Therefore, these indices provide insight into the qualitative properties of circulating lipoproteins and have been associated with an increased risk of progression of subclinical atherosclerosis [26].

In this study, the oxLDL/LDL and oxHDL/HDL ratios were significantly lower in subjects with CAD. This may be explained by the use of lipid-lowering drugs, which can contribute to reducing lipoprotein oxidation, although this is not their primary mechanism of action. These medications exert anti-inflammatory effects and improve endothelial function, thereby promoting a less oxidative environment, which may be reflected in lower ratios of oxidized lipoproteins to total lipoproteins [27,28].

Regarding anthropometric variables, WC was significantly higher among women in the CAD group, consistent with Fang et al., who reported greater WC in their CVD group compared with the non-CVD group (106.5 ± 16.5 vs. 98.5 ± 16.2 cm, *p* < 0.001) [19]. It is well known that abdominal obesity is directly related to insulin resistance; similarly, elevated WC has been associated with an increased risk of mortality in patients with CAD [2,3,29].

Lower PhA values have been associated with chronic inflammation, oxidative stress, and worse clinical outcomes in CAD. Despite its relevance, PhA is not routinely measured in clinical or research settings, making its assessment a valuable measure indicator of cellular health and cardiovascular risk [9,30,31]. In the present study, significantly lower PhA values were observed in men with CAD compared with non-CAD men.

Likewise, individuals with CVD have been shown to present lower PhA values than those without CVD [30]. Studies have associated low PhA values with increased cardiovascular risk, as determined by the Framingham general cardiovascular risk score, and it has even been observed that, for each unit increase in cardiovascular risk, PhA decreases by 1.0 degree in men and 1.2 degrees in women [9,30,32].

An adequate dietary antioxidant intake is necessary, as it represents an effective strategy to reduce oxidative stress and inflammation, limit the progression of atherosclerosis, and modulate cellular signaling pathways. In this sense, the DAI is a validated tool that allows the estimation of the intake of compounds with potential protective effects against oxidative damage at the cellular level [5].

The results showed that among women with CAD classified as having a DAI > −1.2, TC/HDL and LDL/HDL ratios were significantly lower. Additionally, the values of these markers remained within recommended ranges, unlike those observed in the CAD group with low DAI, in which these indices exceeded the recommended ranges. These findings suggest a more favorable lipid profile in women with higher dietary antioxidant intake, which may have important clinical implications given the well-established role of these ratios as predictors of cardiovascular risk. The observed associations may reflect the potential of dietary antioxidants to modulate lipid metabolism, reduce oxidative stress, and improve lipoprotein function, thereby contributing to a less atherogenic profile.

Regarding HDL findings in men classified according to DAI, the results appear somewhat inconsistent, as higher HDL levels were observed in the group with lower DAI. However, this association may have been influenced by potential confounding variables that were not fully accounted for.

According to the linear regression models, a higher DAI was associated with increased HDL concentrations and lower atherogenic indices (TC/HDL and LDL/HDL). Specifically, for each 1-unit increase in the DAI, HDL concentrations increased by 5.8 mg/dL (R^2^ = 0.625). Likewise, the TC/HDL ratio decreased by 0.3 for each unit increase in DAI (R^2^ = 0.625), and the LDL/HDL ratio decreased by 0.2 (R^2^ = 0.506).

These findings are relevant because HDL and these lipid ratios are important predictors of cardiovascular risk and clinical prognosis [2]. Studies have shown that each 1 mg/dL increase in HDL concentration is associated with a 2–3% decrease in CAD risk [33]. The observed association may be partially explained by the antioxidant effect of the diet, which may reduce lipoprotein oxidation, improve endothelial function, and modulate inflammatory responses [34,35].

On the other hand, DAI showed a positive association with PhA in non-CAD subjects. In contrast, this association was not statistically significant in subjects with CAD, which may be explained by the chronic inflammatory and oxidative environment characteristic of the disease, potentially limiting their impact of dietary antioxidant intake on PhA [2,5]. These findings suggest that, in non-CAD subjects, greater dietary antioxidant capacity may support cell membrane integrity and overall antioxidant status. This relation may be reflected in the higher PhA values observed, highlighting the possible role of diet as a modulating factor in cellular health.

The use of PhA and DAI as complementary tools in clinical practice is suggested, given their potential to assess cellular health and oxidative balance in a non-invasive manner. The data obtained reinforce the importance of promoting the consumption of antioxidant-rich foods, which could help improve antioxidant capacity, particularly in individuals with increased cardiometabolic risk, since low values have been associated with poorer outcomes, including in cardiovascular conditions.

The limitations of this study include its analytical cross-sectional design, which precludes the establishment of causal relationships. Additionally, no long-term follow-up was performed to evaluate the impact of dietary patterns on coronary events. Dietary intake was assessed using habitual daily food records, a commonly employed method that may introduce measurement error and affect the accuracy of dietary estimates. Furthermore, the relatively small sample size may limit the generalizability of the findings, reduce statistical power, and increase the risk of overfitting in regression models. Finally, differences in recruitment methods between study groups may have introduced selection bias. Therefore, future studies should consider recruiting non-CAD subjects from the cardiology service of the same hospital.

A key strength of this study is the novel integration of the Dietary Antioxidant Index (DAI), oxidized lipoproteins, and phase angle (PhA), enabling a more comprehensive assessment of cardiovascular risk. Furthermore, this research focuses on Mexican women with CAD, offering clinically relevant insights through the use of innovative, non-invasive markers such as PhA.

The findings of this study provide scientific evidence for further research and suggest potential clinical applications. Further studies are needed to confirm the influence of specific antioxidant-rich dietary interventions on these indicators and to incorporate markers of inflammation and oxidative stress, allowing a broader understanding of the pathophysiological mechanisms. Longitudinal studies evaluating changes in DAI, PhA, and oxidized lipoproteins over time would strengthen the evidence supporting their usefulness as prognostic indicators and clinical monitoring tools.

## 5. Conclusions

The results indicate that, in women with CAD, a higher DAI is associated with a more favorable lipid profile, particularly with higher HDL concentrations, and lower TC/HDL and LDL/HDL ratios. In non-CAD subjects, DAI showed a positive association with PhA. Overall, these findings suggest that DAI influences markers related to cardiovascular health; however, its impact appears to vary according to clinical status and sex. Further longitudinal studies are warranted to assess the effect of dietary patterns on cardiometabolic outcomes.

## Figures and Tables

**Table 1:** Comparison of biochemical and anthropometric variables between CAD and non-CAD groups.

Variables	CAD (*n* = 51)	Non-CAD (*n* = 56)	*p* Value
Biochemical			
TC (mg/dL)	142.4 (130.8–154.0)	184 (173.0–195.0)	<0.001
LDL (mg/dL)	70.7 (60.5–81.0)	108.7 (99.0–118.4)	<0.001
VLDL (mg/dL)	32.8 (27.8–37.8)	31.1 (26.4–35.9)	0.657
HDL (mg/dL)			
M (*n* = 68)	33.6 (30.4–36.8)	41.6 (38.4–44.8)	0.002
W (*n* = 39)	46.5 (38.7–54.3)	49.2 (42.5–56.0)	0.616
TG (mg/dL)	174.2 (146.1–202.3)	154.5 (127.8–181.1)	0.345
TG/HDL ratio			
M (*n* = 68)	5.7 (4.5–7.0)	3.9 (2.6–5.1)	0.053
W (*n* = 39)	3.9 (2.9–5.0)	3.5 (2.6–4.4)	0.517
TC/HDL ratio			
M (*n* = 68)	4.3 (3.8–4.8)	4.4 (3.9–4.9)	0.696
W (*n* = 39)	3.4 (2.8–4.1)	4.1 (3.5–4.6)	0.150
LDL/HDL ratio			
M (*n* = 68)	2.2 (1.8–2.6)	2.6 (2.2–3.0)	0.154
W (*n* = 39)	1.6 (1.2–2.1)	2.4 (1.9–2.8)	0.035
oxLDL (ng/mL)	7362.4 (6322.3–8402.5)	5348.2 (4352.8–6343.6)	0.011
oxHDL (ng/mL)	3.5 (3.2–3.9)	3.3 (3.0–3.7)	0.405
oxLDL/LDL	1.9 (1.8–2.0)	2.4 (2.3–2.5)	<0.001
oxHDL/HDL	5.0 (4.9–5.0)	5.1 (5.0–5.1)	0.005
Glucose (mg/dL)	124.2 (111.3–137.1)	101.7 (89.4–113.9)	0.021
Anthropometric			
BMI (kg/m^2^)	28.9 (27.1–30.7)	28.6 (26.9–30.3)	0.833
WC (cm)			
M (*n* = 68)	97.0 (91.9–102.1)	99.9 (94.8–105.0)	0.458
W (*n* = 38)	94.6 (88.8–100.5)	84.6 (79.7–89.5)	0.016
Body fat (%)			
M (*n* = 60)	27.8 (24.2–31.4)	29.9 (26.8–32.9)	0.416
W (*n* = 29)	41.3 (37.0–45.6)	35.9 (32.9–38.9)	0.051
PhA (°)	6.3 (5.9–6.7)	6.5 (6.2–6.9)	0.436
M (*n* = 59)	6.1 (5.8–6.4)	6.8 (6.5–7.1)	0.005
W (*n* = 28)	6.7 (5.6–7.8)	6.1 (5.3–6.9)	0.365

Values are presented as estimated means with 95% confidence intervals. *p* values were obtained using a univariate general linear model adjusted for age, sex, alcohol consumption, smoking, and physical activity. Oxidized lipoprotein ratios were converted to the natural logarithm (ln). *p* values < 0.05 were considered statistically significant. CAD: coronary artery disease; M: men; W: women; TC: total cholesterol; TG: triglycerides; LDL: low-density lipoprotein cholesterol; HDL: high-density lipoprotein cholesterol; VLDL: very-low-density lipoprotein cholesterol; oxLDL: oxidized low-density lipoprotein; oxHDL: oxidized high-density lipoprotein; BMI: body mass index; WC: waist circumference; PhA: phase angle. Units: TC, LDL, VLDL, HDL, TG, and glucose are expressed in mg/dL (milligrams per deciliter); oxLDL and oxHDL in ng/mL (nanograms per milliliter); and BMI in kg/m^2^ (kilograms per square meter).

**Table 2:** Comparison of biochemical and anthropometric variables between study groups classified according to the median DAI.

Variables	CAD		*p-*Value	Non-CAD		*p-*Value
	DAI < −1.2 (*n* = 26)	DAI > −1.2 (*n* = 25)		DAI < −0.4 (*n* = 28)	DAI > −0.4 (*n* = 28)	
Biochemical						
TC (mg/dL)	150.1 (133.6–166.5)	135.9 (119.2–152.7)	0.236	186.9 (175.3–198.5)	179.8 (168.2–191.4)	0.394
LDL (mg/dL)	79.7 (64.5–94.9)	64.8 (49.3–80.3)	0.179	112.3 (102.3–122.3)	102.0 (92.0–112.0)	0.153
HDL (mg/dL)						
M (*n* = 68)	37.3 (33.3–41.3)	29.4 (25.4–33.4)	0.009	41.8 (36.9–46.6)	42.1 (37.7–46.4)	0.927
W (*n* = 39)	38.3 (26.0–50.6)	53.8 (40.8–66.9)	0.089	50.9 (43.0–58.8)	48.6 (39.0–58.2)	0.715
VLDL (mg/dL)	32.8 (26.5–39.2)	33.0 (26.5–39.5)	0.973	29.4 (22.9–36.0)	32.6 (26.0–39.1)	0.500
TG/HDL ratio						
M (*n* = 68)	5.2 (3.3–7.1)	5.8 (3.9–7.7)	0.642	3.6 (2.0–5.1)	4.5 (3.1–5.9)	0.365
W (*n* = 39)	4.6 (3.2–6.0)	3.8 (2.3–5.2)	0.383	3.4 (2.2–4.5)	3.0 (1.7–4.4)	0.724
TC/HDL ratio						
M (*n* = 68)	3.8 (3.1–4.5)	4.7 (4.0–5.4)	0.089	4.6 (4.0–5.1)	4.4 (3.9–4.9)	0.663
W (*n* = 39)	4.5 (3.9–5.1)	2.8 (2.2–3.5)	0.001	3.9 (3.4–4.5)	3.7 (3.0–4.4)	0.631
LDL/HDL ratio						
M (*n =* 68)	1.8 (1.2–2.5)	2.5 (1.9–3.1)	0.139	2.9 (2.4–3.3)	2.5 (2.1–2.9)	0.217
W (*n* = 39)	2.6 (2.1–3.1)	1.1 (0.5–1.6)	0.001	2.2 (1.8–2.7)	2.1 (1.6–2.6)	0.622
TG (mg/dL)	181.5 (141.9–221.2)	164.7 (124.3–205.1)	0.557	147.1 (114.3–179.9)	163.5 (130.7–196.3)	0.485
oxLDL (ng/mL)	6535.0 (5320.8–7749.1)	7636.4 (6371.1–8901.7)	0.219	5644.1 (4329.6–6958.6)	5604.1 (4238.4–6969.7)	0.967
oxHDL (ng/mL)	3.5 (3.1–4.0)	3.5 (3.1–4.0)	0.948	3.3 (2.9–3.7)	3.4 (3.1–3.8)	0.557
oxLDL/LDL	2.0 (1.9–2.1)	1.8 (1.7–2.0)	0.054	2.3 (2.25–2.53)	2.3 (2.2–2.5)	0.939
oxHDL/HDL	5.0 (4.9–5.0)	5.0 (4.9–5.0)	0.701	5.1 (5.0–5.2)	5.1 (5.0–5.1)	0.513
Glucose (mg/dL)	138.2 (114.3–162.0)	119.8 (95.5–144.1)	0.288	96.9 (91.2–102.6)	97.5 (91.8–103.2)	0.875
Anthropometric						
BMI (kg/m^2^)	28.4 (26.2–30.5)	30.2 (28.0–32.4)	0.248	28.8 (26.6–30.9)	27.7 (25.5–29.9)	0.496
WC (cm)						
M (*n* = 68)	94.1 (87.1–101.1)	102.6 (95.6–109.6)	0.102	97.2 (90.4–104.0)	99.8 (93.7–105.8)	0.567
W (*n* = 38)	98.1 (90.9–105.4)	96.3 (88.0–104.6)	0.729	85.6 (79.0–92.1)	78.4 (70.4–86.5)	0.182
Body fat (%)						
M (*n* = 60)	27.4 (21.5–33.4)	30.1 (24.6–35.6)	0.517	30.1 (26.3–33.8)	28.2 (24.9–31.6)	0.465
W (*n* = 29)	44.9 (39.6–50.2)	36.0 (27.2–44.8)	0.091	36.5 (32.6–40.3)	34.0 (29.4–38.6)	0.414
PhA (°)	6.2 (5.5–6.9)	6.0 (5.2–6.7)	0.615	6.5 (6.2–6.8)	6.8 (6.6–7.1)	0.076
M (*n* = 59)	5.7 (5.2–6.2)	5.9 (5.4–6.4)	0.476	6.8 (6.5–7.2)	7.2 (6.9–7.5)	0.096
W (*n* = 28)	7.0 (3.7–10.3)	6.5 (1.0–12.1)	0.861	5.9 (5.4–6.4)	6.1 (5.6–6.6)	0.610

Values are presented as estimated means with 95% confidence intervals. *p* values were obtained using a univariate general linear model adjusted for age, sex, alcohol consumption, smoking, and physical activity. Oxidized lipoprotein ratios were converted to the natural logarithm (ln). *p* values < 0.05 were considered statistically significant. CAD: coronary artery disease; DAI: dietary antioxidant index; M: men; W: women; TC: total cholesterol; TG: triglycerides; LDL: low-density lipoprotein cholesterol; HDL: high-density lipoprotein cholesterol; VLDL: very-low-density lipoprotein cholesterol; oxLDL: oxidized low-density lipoprotein; oxHDL: oxidized high-density lipoprotein; BMI: body mass index; WC: waist circumference; PhA: phase angle. Units: TC, LDL, VLDL, HDL, TG, and glucose are expressed in mg/dL (milligrams per deciliter); oxLDL and oxHDL in ng/mL (nanograms per milliliter); and BMI in kg/m^2^ (kilograms per square meter).

**Table 3:** Association between DAI and biochemical variables in women with CAD.

Women with CAD (*n* = 17)	R^2^	B (95% CI)	*p* Value
HDL (mg/dL)	0.625	5.8 (2.9–8.7)	0.001
TC/HDL ratio	0.625	−0.3 (−0.5–−0.1)	0.006
LDL/HDL ratio	0.506	−0.2 (−0.5–−0.07)	0.012

Linear regression models were adjusted for age, alcohol consumption, smoking, and physical activity. *p* values < 0.05 were considered statistically significant. CAD: coronary artery disease; HDL: high-density lipoprotein cholesterol; TC: total cholesterol; LDL: low-density lipoprotein cholesterol. Dependent variable: HDL, TC/HDL ratio, and LDL/HDL ratio. Independent variable: DAI.

**Table 4:** Association between DAI and PhA in non-CAD subjects.

Non-CAD Subjects (*n* = 51)	R^2^	B (95% CI)	*p* Value
PhA	0.501	0.07 (0.02–0.1)	0.006

Linear regression models were adjusted for age, sex, alcohol consumption, smoking, and physical activity. *p* values < 0.05 were considered statistically significant. CAD: coronary artery disease; PhA: phase angle. Dependent variable: PhA. Independent variable: DAI.

## Data Availability

The data presented in this study are available from the corresponding author upon reasonable request, subject to privacy restrictions.

## Abbreviations

The following abbreviations are used in this manuscript:

BFP	Body fat percentage
BMI	Body mass index
CAD	Coronary artery disease
CVD	Cardiovascular diseases
DAI	Dietary antioxidant index
ELISA	Enzyme-linked immunosorbent assay
FRAP	Ferric-reducing antioxidant po
HDL	High-density lipoprotein cholesterol
LDL	Low-density lipoprotein
NSTEMI	Non-ST segment elevation myocardial infarction
oxHDL	Oxidized high-density lipoprotein
oxLDL	Oxidized low-density lipoprotein
PhA	Phase angle
PUFA	Polyunsaturated fatty acid
ROS	Reactive oxygen species
STEMI	ST-segment elevation myocardial infarction
TC	Total cholesterol
TEAC	Trolox-equivalent antioxidant capacity
TG	Triglycerides
TRAP	Total radical-trapping antioxidant parameters
VLDL	Very-low-density lipoprotein
